# HSCCC-based strategy for preparative separation of *in vivo* metabolites after administration of an herbal medicine: *Saussurea laniceps*, a case study

**DOI:** 10.1038/srep33036

**Published:** 2016-09-13

**Authors:** Tao Yi, Lin Zhu, Guo-Yuan Zhu, Yi-Na Tang, Jun Xu, Jia-Yi Fan, Zhong-Zhen Zhao, Hu-Biao Chen

**Affiliations:** 1 School of Chinese Medicine, Hong Kong Baptist University, Kowloon Tong, Hong Kong Special Administrative Region, China; 2 Institute of Research and Continuing Education (Shenzhen), Hong Kong Baptist University, Shenzhen, China; 3 State Key Laboratory of Quality Research in Chinese Medicine, Macau University of Science and Technology, Taipa, Macau Special Administrative Region, China

## Abstract

This paper reports a novel strategy based on high-speed counter-current chromatography (HSCCC) technique to separate *in vivo* metabolites from refined extract of urine after administration of an herbal medicine. *Saussurea laniceps* (SL) was chosen as a model herbal medicine to be used to test the feasibility of our proposed strategy. This strategy succeeded in the case of separating four *in vivo* metabolites of SL from the urine of rats. Briefly, after oral administration of SL extract to three rats for ten days (2.0 g/kg/d), 269.1 mg of umbelliferone glucuronide (**M1**, purity, 92.5%), 432.5 mg of scopoletin glucuronide (**M2**, purity, 93.2%), 221.4 mg of scopoletin glucuronide (**M3**, purity, 92.9%) and 319.0 mg of scopoletin glucuronide (**M4**, purity, 90.4%) were separated from 420 mL of the rat urine by HSCCC using a two-phase solvent system composed of methyl *tert*-butyl ether–*n*-butanol–acetonitrile–water (MTBE–*n*-BuOH–ACN–H_2_O) at a volume ratio of 10:30:11:49. The chemical structures of the four metabolites, **M1** to **M4,** were confirmed by MS and ^1^H, ^13^C NMR. As far as we know, this is the first report of the successful separation of *in vivo* metabolites by HSCCC after administration of an herbal medicine.

After administration of drugs to humans, the drugs are eliminated from the body in two forms: the original drugs and/or drug-derived metabolites. In the development of new drugs, study of the metabolism of the candidate new drugs has been emphasized. Currently, investigation of the differences between the original drugs and their related metabolites in terms of their biological activity, as well as prediction of the potential pharmacological effects and toxic effects of the metabolites have become important parts of the assessment to determine whether a candidate drug/compound has value enough for further research and development^1^,^2^. Unlike synthetic drugs, an herbal medicine (HM) is a synergistic system comprising multiple components. Because of the complicated chemical composition of an HM, a number of compounds may enter the circulation and then be converted into even more metabolites, which make the clarification of the bio-transformed metabolites very challenging^3^,^4^. Most current reports on the identification of HM metabolites have been based on using online LC-DAD, LC-MS, LC-MS/MS and GC-MS^5^,^6^,^7^,^8^,^9^,^10^. Unfortunately, these methods can give only tentative identifications; unambiguous structural elucidation still depends on the NMR information of the isolated metabolites^11^,^12^,^13^,^14^. Furthermore, isolation of these metabolites has been difficult, yet sufficient pure metabolites are essential to further pharmacological and toxicological studies. Therefore, how to obtain *in vivo* metabolites with satisfactory purity and yield remains the key step to in-depth study of metabolites for understanding the efficacy and/or toxicity of traditional HM.

The excreta of animals that have been administered with HM have been the main sources for *in vivo* metabolites separation, besides the products of microbial transformation, chemical structure modification, original plants and plant tissue culture^15^. However, lack of a suitable separation method has obstructed the acquisition of *in vivo* metabolites for a long time. Conventional separation techniques typically used include preparative thin layer chromatography^16^, preparative high-performance liquid chromatography (HPLC)^17^, and column chromatography with various support materials, such as silica gel^11^, macroporous resin^18^, polyamide^18^,^19^, Sephadex LH 20^20^, and octadecylsilane (ODS)^18^,^20^. Among these techniques, HPLC is a popular and easy separation technique with high efficiency, high throughput, high yield and suitable for complex matrices. All of these conventional techniques, however, suffer from the intrinsic weakness of sample loss due to irreversible adsorption of sample onto the solid support matrix.

High-speed counter-current chromatography (HSCCC) is an alternative to HPLC, and developed in the early 1980s by Yoichiro Ito at the National Institutes of Health (Bethesda, MD, USA)^21^,^22^. HSCCC is a support-free liquid-liquid partition chromatography technique, and based on the principle that solutes that have a different relative solubility in two immiscible liquid phases. Solute separation using HSCCC involves the use of a support-free liquid stationary phase, a liquid mobile phase, and a centrifugal force field. The centrifugal force field retains the liquid stationary phase, while the liquid mobile phase is pushed through it. As a result of static partition and dynamic elution under the influence of the continuously flowing mobile phase, the different solutes separate^23^.

Compared with other conventional column chromatography methods, such as HPLC, HSCCC offers the following three important advantages^24^,^25^,^26^,^27^: 1) high recovery almost without sample loss. As both phases are liquid, HSCCC eliminates irreversible adsorption and degradation of samples on solid supports such as silica gel or ODS; therefore the sample can be totally recovered; 2) high yield and high efficiency. Higher amounts of sample introduced into the HSCCC apparatus and easy scale-up from milligrams to grams in preparative separation result in a higher yield and process-efficiency. Meanwhile, lower costs of ownership and consumables lead to greater cost-efficiency for HSCCC; 3) a wide range of applications. Theoretically, HSCCC can be applied to separate samples with any polarity, because the composition and proportion of the two liquid phases system may be infinite. These advantages make HSCCC more powerful and more widely used than HPLC in the separation and purification of different kinds of natural products^28^,^29^,^30^,^31^,^32^. However, as far as we know, no study has been reported on the use of HSCCC for purification of *in vivo* metabolites after oral administration of HM.

Therefore, in the present work, we propose a strategy based on HSCCC to separate the metabolites of an HM after it is administered to rats. The strategy consists of three key steps: 1) firstly, comprehensively screen the various biofluids (plasma, urine, feces, etc.) after administration of an herb to rats by LC-MS, etc.; 2) secondly, separate the target metabolites from the desirable biofluids by HSCCC; 3) thirdly, elucidate the structure of the obtained metabolites by NMR, etc. This strategy was used with the Tibetan “Snow Lotus” herb (*Saussurea laniceps*) as a model herb to test its feasibility.

Tibetan “Snow Lotus” herb (*Saussurea laniceps*, SL) was selected as the test herb because our lab is very familiar with this herb’s chemistry and because further pharmacological development of this herb is particularly hindered by failure to purify its metabolites. In previous studies, we have already explored the chemical composition of SL by using HPLC^33^,^34^, and investigated the anti-inflammatory effects by using animal models^35^,^36^. Moreover, we have conducted metabolic profiles analysis by LC-MS after SL oral administration to rats^37^. In order to evaluate the biological effects of the newly generated metabolites through pharmacological trials in the future, it is necessary to obtain reasonably large quantities of highly purified compounds. All the previous studies lay a solid foundation for this *in vivo* metabolite separation of SL.

## Results and Discussion

### Screening and selection of the biofluids for metabolite separation

**Figure 1:**
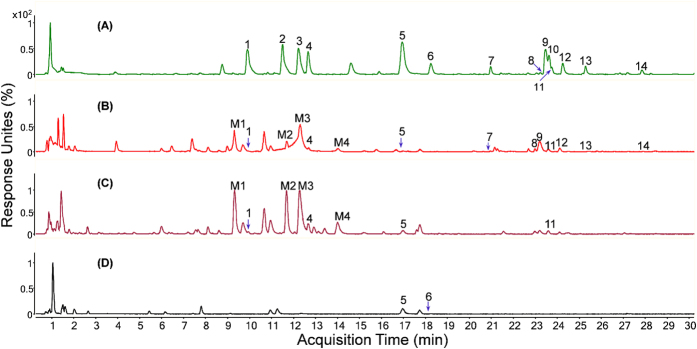
UPLC-MS chromatograms of biofluids after oral administration of SL extract in negative ion mode. (**A**) SL extract, (**B**) rat plasma, (**C**) urine and (**D**) feces.

**Figure 2:**
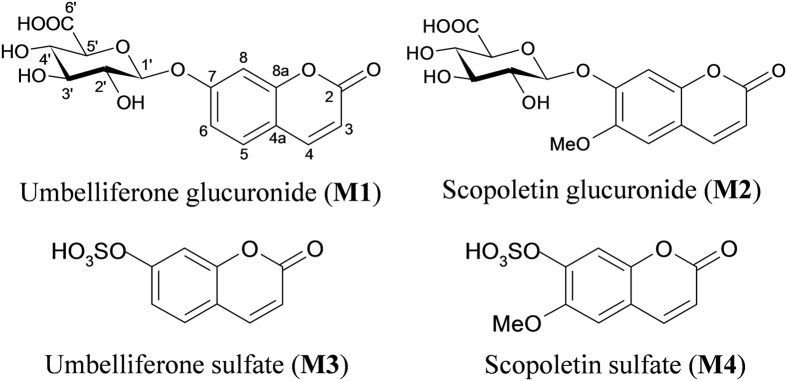
The chemical structures of the target metabolites.

The sample solutions of SL extract (A), rat plasma (B), urine (C) and feces (D) were injected into the LC-MS system, and the results are shown in Fig. 1. Based on previous study^37^, the peaks were attributed to skimming (**1**), chlorogenic acid (**2**), scopolin (**3**), syringoside (**4**), umbelliferone (**5**), scopoletin (**6**), isoquercitroside (**7**), 3,4-dicaffeoylquinic acid (**8**), 1,5-dicaffeoylquinic acid (**9**), 3,5-dicaffeoylquinic acid (**10**), apigenin 7-*O*-α-L-rhamnosyl- (1→2)-β-D-glucoside (**11**), apigenin 7-*O*-β-D-glucoside (**12**) and 4,5-dicaffeoylquinic acid (**13**). For peak 14, the identification is in progress. Moreover, four new metabolites (**M1**, **M2**, **M3** and **M4**) were tentatively identified to be umbelliferone glucuronide (**M1**), scopoletin glucuronide (**M2**), umbelliferone sulfate (**M3**), and scopoletin sulfate (**M4**) by comparing their high resolution MS (HRMS) spectrum with the original compounds, umbelliferone (**5**) and scopoletin (**6**). The tentative elucidation process is shown in our previous study^37^, and their structures are shown in Fig. 2.

**M1**, **M2**, **M3** and **M4** were chosen as the target metabolites. Although these metabolites exist in both blood and urine, urine was chosen as the more desirable source for metabolite separation because: 1) sufficient collection causes no harm to animals; 2) urine has more metabolites and fewer endogenous interfering substances.

### Refinement of rat urine sample

Urine is an aqueous solution of water (>95%) with a small proportion of inorganic salts (ca. 3%) and other dissolved compounds. The maximum sample loading of the TBE-1000A CCC machine is 80 mL for each injection, but our volume of rat urine (420 mL) is well beyond that maximum. Repeated HSCCC operation, at 80 mL per time, is prohibitively time-consuming. Furthermore, the inorganic salts in urine will precipitate when meeting with organic solvent, resulting in tube blockage during the HSCCC separation process. Therefore, in order to efficiently use HSCCC, the target metabolites need to be enriched and inorganic salts removed. To achieve this goal, freeze-drying was used to remove the excessive water, methanol was used to precipitate inorganic salts and extract the metabolites, and the mobile phase solution was used to re-dissolve the refined urine sample.

Specifically, the pooled rat urine samples of 420 mL were lyophilized to obtain urine residue (15.9 g, yield 3.8%, w/v). And then, the freeze-dried urine residue was extracted in 150 mL of methanol by means of sonication, three times. The combined extract was evaporated to obtain refined urine (4.3 g, yield 1.0%, w/v). Finally, the refined urine extract (4.3 g) obtained from original urine sample of 420 mL was re-dissolved in 80 mL of the mobile phase solution. Through this procedure, the contents of target metabolites in the refined urine sample and the loading capacity of a single HSCCC separation were increased more than 5-fold when compared with untreated urine samples.

### Optimization of HSCCC conditions

In HSCCC, various parameters, including the type and composition of the two-phase solvent system, flow rate of the mobile phase and revolution speed, affect separation efficacy. Among the parameters, selection of the two-phase solvent system is generally considered to be the most important. The partition coefficient (*K*), the ratio of solute distributed between the mutually equilibrated two solvent phases, is the most crucial factor in selection of such a solvent system, and the suitable *K* value for HSCCC should be around 0.5–2.0 to obtain an acceptable separation outcome and a practicable separation time. A smaller *K* value elutes the solute closer to the solvent front with lower resolution while a larger *K* value tends to give better resolution but broader, more dilute peaks due to a longer elution time.

According to the golden guidelines for HSCCC^38^, two solvent systems are mainly used for HSCCC, namely *n*-hexane–ethyl acetate–methanol–water solvent system and methyl *tert*-butyl ether (MTBE) –*n*-butanol (*n*-BuOH) –acetonitrile (ACN) –water (H_2_O) solvent system. The former is usually suitable for the separation of components with medium polarity, while the latter is suitable for the separation of polar components. Given that our target compounds in the present research are polar, MTBE–*n*-BuOH–ACN–H_2_O was chosen as the two-phase solvent system. To further optimize the composition of MTBE–*n*-BuOH–ACN–H_2_O system, partition coefficients (*K*) of the four metabolites in the two-phase solvent systems with different ratios of volume were evaluated. Our experimental design and results are summarized in Table 1. The results indicate that the solvent system composed of MTBE–*n*-BuOH–ACN–H_2_O at a volume ratio of 2:2:1:5 and 0:36:15:49 (v/v/v/v) had a small *K* value and the solvent systems at volume ratios of 5:30:10:53 and 3:32:10:55 (v/v/v/v) had large *K* values, which were not suitable for the isolation and separation. Thus, the solvent system composed of MTBE–*n*-BuOH–ACN–H_2_O at a volume ratio of 10:30:11:49 (v/v/v/v) was selected for purification in the present research.

Apart from a suitable two-phase solvent system, other parameters that may affect the isolation results in HSCCC include column temperature, revolution speed and flow rate of the mobile phase. In the present paper, different column temperatures (20, 25, and 30 ^o^C), different revolution speeds (400, 450, 500 and 550 rpm), different flow rates (5.0, 8.0 and 10.0 mL/min) of the mobile phase of the selected system were optimized by single-factor experimental design according to the manufacturer’s protocol. In brief, when we chose column temperatures, the revolution speed and flow rate were fixed at 450 rpm and 8.0 mL/min, respectively; after the optimized temperature of 25 ^o^C was found, we chose revolution speeds based on the optimized temperature (25 ^o^C) and a fixed flow rate of 8.0 mL/min; after the optimized speed of 500 rpm was confirmed, flow rates were chosen based on the optimized temperature of 25 ^o^C and optimized revolution speed of 500 rpm. Finally, the most suitable isolation conditions were optimized as follows: the two-phase solvent system was composed of MTBE–*n*-BuOH–ACN–H_2_O (10:30:11:49, v/v/v/v), the lower phase was used as the mobile phase at 8.0 mL/min, the column temperature and revolution speed were set at 25 ^o^C and 500 rpm. Under the optimized conditions, four peaks were isolated and collected within 420 min.

### HSCCC separation outcomes

**Figure 3:**
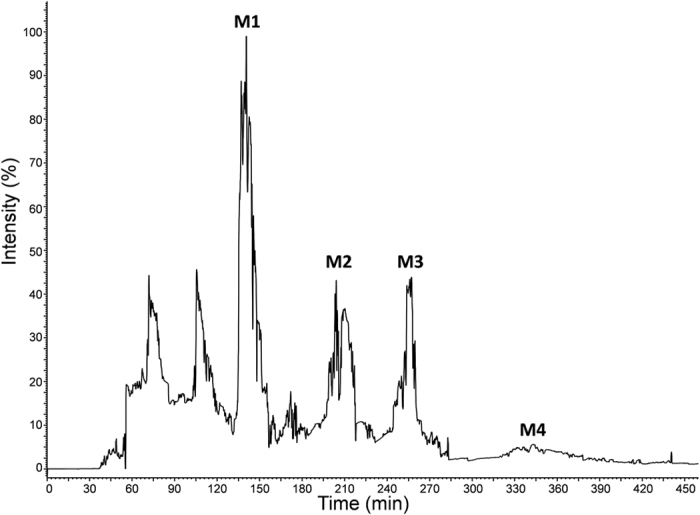
HSCCC chromatogram of the refined urine sample after SL administration. Solvent system: MTBE–*n*-BuOH–ACN–H_2_O (10:30:11:49, v/v/v/v). Detection wavelength was 280 nm. Sample loading was 4.3 g of refined urine extract in 80 mL of lower phase. Rotation speed was 500 rpm. The temperature of separation columns was maintained at 25 ^o^C, and the flow rate of the mobile phase was 8.0 mL/min. Peak 1: Umbelliferone glucuronide (**M1**); Peak 2: Scopoletin glucuronide (**M2**); Peak 3: Umbelliferone sulfate (**M3**); Peak 4: Scopoletin sulfate (**M4**).

**Figure 4:**
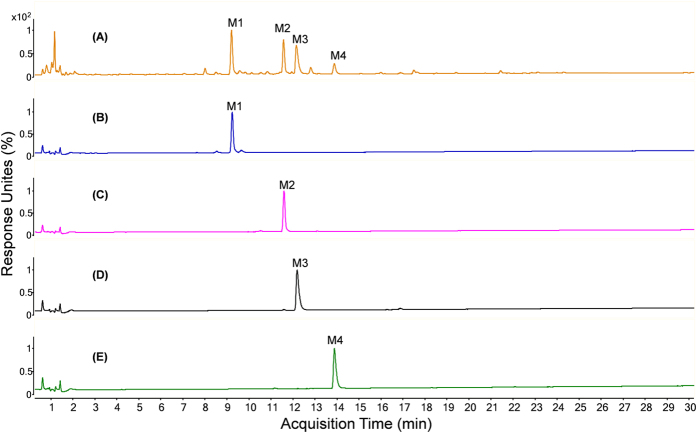
UPLC-UV chromatogram of (**A**) rat urine after SL administration, (**B**) metabolite M1, (**C**) M2, (**D**) M3, and (**E**) M4 obtained by HSCCC separation. Column: Waters HSS C_18_ column (1.7 μm, 2.1 mm × 100 mm, Waters Corp.) with a VanGuard^TM^ pre-column (HSS C_18_, 1.7 μm, 2.1 mm × 5 mm); mobile phase: 0.1% formic acid in water (a) and 0.1% formic acid in acetonitrile (b) using a gradient program of 3% (b) in 0–2 min, 3–15% (b) in 2–14 min, 15–20% (b) in 14–22 min and 20–45% (b) in 22–30 min; flow rate: 0.3 mL/min; detection wavelength: 280 nm.

As illustrated in Fig. 3, four fractions (**M1**, 130–150 min, 269.1 mg; **M2**, 190–220 min, 432.5 mg; **M3**, 240–270 min, 221.4 mg; **M4**, 315–400 min, 319.0 mg) were separated from 4.3 g of refined rat urine extract (equal to 420 mL of untreated urine sample) after HSCCC separation. For the recovery evaluation, 48.6 mg of **M1** (recovery, 97.2%), 47.3 mg of **M2** (recovery, 94.6%), 48.2 mg of **M3** (recovery, 96.4%) and 46.8 mg of **M4** (recovery, 93.6%) were separated by HSCCC from the spiked urine sample. The purities of the obtained metabolites **M1**–**M4** were determined to be 92.5%, 93.2%, 92.9% and 90.4%, respectively, by NMR internal standard method. These results clearly demonstrate that HSCCC provides excellent separation of *in vivo* metabolites with satisfactory purity and yield from rat urine after oral administration of SL extract (Fig. 4).

### Chemical structure elucidation

The chemical structure identification was carried out by UV, MS, and NMR as follows:

Umbelliferone glucuronide (**M1**), white amorphous powder; UV (MeOH) λ_*max*_: 198, 215, 320 nm; HR-ESI-MS m/z: 339.0713 [*M* + H]^+^ (calculated for C_15_H_15_O_9_ [*M* + H]^+^, 339.0711); MS^2^ yielded ions at m/z 163.0389 ([*M* + H]^+^-176, loss of glucuronic acid). NMR and MS spectra for all the metabolites are shown in Supplementary Information. NMR data and assignments of carbon and proton signals are summarized in Table 2. The above data are in agreement with the literature^39^.

Scopoletin glucuronide (**M2**), white amorphous powder; UV (MeOH) λ_*max*_: 203, 225, 338 nm; HR-ESI-MS m/z: 369.0817 [*M* + H]^+^ (calculated for C_16_H_17_O_10_ [*M* + H]^+^, 369.0816); MS^2^ yielded ions at m/z 193.0490 ([*M* + H]^+^-176, loss of glucuronic acid). NMR data and assignments of carbon and proton signals are shown in Table 2. Compared with **M1**, the minor change of **M2** with substitution of 6-H with 6-OMe was confirmed by the disappearance of the signal of 6-H at *δ*_H_ 7.12 and the generation of the singlet methyl signal at *δ*_H_ 3.91 as well as the carbon signal of methoxyl at *δ*_C_ 57.0. Meanwhile, the signal of 6-C adjacent to methoxyl was upshifted from *δ*_C_ 115.2 to *δ*_C_ 148.3. This replacement was also confirmed by the change of molecular weight of **M2** (C_16_H_16_O_10_) which was 30 mass units (OCH_2_) less than that of **M1** (C_15_H_14_O_9_).

Umbelliferone sulfate (**M3**), white amorphous powder; UV (MeOH) λ_*max*_: 198, 213, 312 nm; HR-ESI-MS m/z: 242.9955 [*M* + H]^+^ (calculated for C_9_H_7_O_6_S [*M* + H]^+^, 242.9958); MS^2^ yielded ions at m/z 163.0382 ([*M* + H]^+^-80, loss of a SO_3_ fragment). NMR data and assignments of carbon and proton signals are shown in Table 2. The above data are in agreement with the literature^39^.

Scopoletin sulfate (**M4**), white amorphous powder; UV (MeOH) λ_*max*_: 205, 218, 338 nm; HR-ESI-MS m/z: 273.0062 [*M* + H]^+^ (calculated for C_10_H_9_O_7_S [*M* + H]^+^, 273.0063); MS^2^ yielded ions at m/z 193.0486 ([*M* + H]^+^-80, loss of a SO_3_ fragment). NMR data and assignments of carbon and proton signals are shown in Table 2. The overall ^1^H and ^13^C NMR data of **M4** closely resembled that of **M3**, except that the proton signal of 6-H at *δ*_H_ 7.26 was missing and instead a new singlet methyl signal at *δ*_H_ 3.89 as well as the carbon signal of methoxyl at *δ*_C_ 57.0 appeared. This change was deduced as the replacement of 6-H in **M3** with 6-OMe in **M4**, which was confirmed by the upshift of C-6 from *δ*_C_ 118.9 to *δ*_C_ 150.2 as well as the molecular weight difference of 30 mass units (OCH_2_) between **M3** (C_9_H_6_O_6_S) and **M4** (C_10_H_8_O_7_S).

In conclusion, this paper describes the first success using HSCCC for preparative separation of *in vivo* metabolites after administration of an herbal extract to animals; both high purity and a remarkable yield were achieved. After oral administration of SL extract to three rats for ten days (2.0 g/kg/d), 269.1 mg of umbelliferone glucuronide (**M1**, purity, 92.5%), 432.5 mg of scopoletin glucuronide (**M2**, purity, 93.2%), 221.4 mg of scopoletin glucuronide (**M3**, purity, 92.9%) and 319.0 mg of scopoletin glucuronide (**M4**, purity, 90.4%) were separated from 420 mL of the rat urine by HSCCC. Recoveries of the HSCCC obtained from the metabolites spiked urine sample were found to be 93.6–97.2%. The present case study demonstrates that the proposed HSCCC-based strategy is feasible and efficient, and that HSCCC is a powerful tool for the separation and purification of *in vivo* metabolites. This novel strategy can be particularly valuable for preparing pure metabolites in the large quantities needed for laboratory and clinical studies in the research and development of new drugs.

## Methods

### Materials and reagents

*Saussurea laniceps* (SL) was collected in Tibet (China) in September, 2014. The material was identified according to macroscopic and microscopic features^40^. Voucher specimens (No. MT-11) were deposited in the Chinese Medicines Center, Hong Kong Baptist University.

Ethanol was purchased from Merck (Darmstadt, Germany). Acetonitrile (ACN) and formic acid of HPLC grade, *n*-butanol, ethyl acetate, methanol, chloroform of analytical grade were purchased from Lab-scan (Bangkok, Thailand). Hydroquinone with a purity of 99.5% was purchased from Phytomarker Ltd. (Tianjin, China). Methyl *tert*-butyl ether (MTBE) and other chemicals of analytical grade were purchased from Sigma (St. Louis, MO). The water used in the experiments was collected from a Mili-Q ultrapure water system (Bedford, MA, USA).

### Collection of biofluids after oral administration of SL extract to rats

SL extract was prepared as in our previous study^36^. Briefly, herbal sample powder (0.5 kg) was extracted with 50% ethanol by percolation. The combined leachates were evaporated to remove ethanol at reduced pressure in a rotary evaporator and then to obtain the SL extract (76.5 g, yield 15.3%, w/w). For administration to animals, the dried extract (25.0 g) was suspended in 200 mL of aqueous carboxy methylcellulose (1%, w/v).

Three male Sprague–Dawley rats weighing 220–250 g were purchased from the Laboratory Animal Services Center, the Chinese University of Hong Kong, Hong Kong. All experimental protocols were approved by the Committee on the Use of Human & Animal Subjects in Teaching and Research of Hong Kong Baptist University, in accordance with the Animals Ordinance (Department of Health, Hong Kong). The rats were housed individually in metabolic cages with temperature of 23 ± 1 ^o^C, humidity of 60 ± 5%, and 12 h dark – light cycle. SL extract (2.0 g/kg) was orally administered to each of three rats for ten consecutive days. The retro-orbital blood samples (c.a. 0.5 mL) were withdrawn via the cannular at 20 min post-dosing of SL extract. Rat feces samples were collected from 0 to 24 h after oral administration of SL extract.

Rat urine samples (c.a. 420 mL) were collected for ten days after the oral administration of SL extract. The combined urine was filtered with qualitative filter paper (No. 1, ø 150 mm, Advantec, Tokyo, Japan) and then lyophilized with a freeze-dry system to obtain urine extract (15.9 g, yield 3.8%, w/v). The freeze-dried urine residue was extracted in 150 mL of methanol by means of sonication at room temperature for 30 min, and the operation was repeated twice. The combined extract was evaporated to obtain refined urine sample (4.3 g, yield 1.0%, w/v) under vacuum (*T* = 40 ^o^C). The blood and feces samples were treated as prescribed in our previous report^37^.

### Screening the metabolic biofluids and characterizing the target metabolites by UPLC-MS

The qualitative analysis of biofluids was performed with ultrahigh performance liquid chromatography coupled with quadrupole time-of-flight high resolution mass spectrometry (UPLC-QTOF-MS, Agilent Technologies, G6540A). The column used was a Waters HSS C_18_ column (1.7 μm, 2.1 mm × 100 mm, Waters Corp.) with a VanGuard^TM^ pre-column (HSS C_18_, 1.7 μm, 2.1 mm × 5 mm). The mobile phase consisted of 0.1% formic acid in water (A) and 0.1% formic acid in acetonitrile (B) using a gradient program of 3% (B) in 0–2 min, 3–15% in 2–14 min, 15–20% (B) in 14–22 min and 20–45% (B) in 22–30 min. The solvent flow rate was 0.3 mL/min, the column temperature was set to 40 ^o^C and the detection wavelength was 280 nm. For the mass spectrometer conditions, an electrospray ionization (ESI) source was used in negative ion mode, and the desolvation temperature was set at 300 ^o^C, desolvation gas (N_2_) 8 L/min, fragmentor voltage 150 V, capillary voltage 4.5 kV. Sheath gas was set at 350 ^o^C and 9 L/min. All the results were analyzed by Aglient MassHunter Workstation with Quantitative Analysis (Q-TOF) B.04.00 software.

### HSCCC apparatus

The preparative HSCCC was carried out with a Model TBE-1000A high-speed counter-current chromatograph (Tauto Biotech, Shanghai, China) equipped with a 1000 mL coil column made of PTFE tubing (i.d. of the tubing = 3.0 mm, total volume = 1000 mL) and a 80 mL sample loop. The *β*-value of the column varied from 0.59 at the internal layer to 0.75 at the external layer. The rotation speed of the apparatus can be regulated with a speed controller in the range between 0 and 600 rpm. The HSCCC system was connected to a Waters Prep 150 LC system consisting of a 2545 binary gradient pump, a 2489 UV/Visible detector operating at 280 nm, a fraction collector III and a MassLynx v4.1 chromatography workstation (Waters Corp., Milford, MA, USA).

### Preparation and selection of the solvent system

Two-phase solvent system used in the present study was prepared by mixing MTBE–*n*-BuOH–ACN–H_2_O in various proportions (2:2:1:5, 0:36:15:49, 10:30:11:49, 5:30:10:53, or 3:32:10:55, v/v). The solvent mixture was thoroughly equilibrated in a separation funnel at room temperature, and the two phases were separated shortly before use. The optimization of solvent systems was evaluated by UPLC according to the partition coefficients (0.5 < *K *< 2.0) in a series of solvent systems as follows: About 1.0 mg refined urine extract was added to the test tubes, and then 2 mL of each phase of a pre-equilibrated two-phase solvent system was added and thoroughly mixed. Each test tube was rigorously shaken for several minutes and left to stand at room temperature until equilibrium was attained. Then 2 μL of the upper and lower phases were analysed by UPLC at detection wavelength of 280 nm; the gradients of the mobile phase are as same as those of UPLC-MS. The partition coefficient (*K*) is defined as *A*_upper_/*A*_lower_, where *A*_upper_ and *A*_lower_ are the UPLC peak areas of the target metabolites in the upper and lower phases, respectively.

### Preparation of sample solution

The sample solution for isolation and purification by HSCCC was prepared by dissolving the refined urine extract (4.3 g) in the lower phase (80 mL).

### HSCCC separation procedure

The coil column was first entirely filled with the upper phase of the solvent system at a flow rate of 20 mL/min. Then the apparatus was rotated at 500 rpm, while the lower phase was pumped into the column at a flow rate of 8.0 mL/min. After the upper phase emerged and the volume stopped changing, the liquid-liquid equilibrium was established in the column. About 80 mL sample solution containing 4.3 g of the urine extract was injected through the injection value. The effluent was continuously monitored with UV detector at 280 nm, and peak fractions were collected according to the chromatogram. The temperature of the apparatus was maintained at 25 ^o^C. The recovery of the HSCCC procedure was evaluated by comparison with spiked samples. Spiked samples were prepared by adding 50 mg of each isolated metabolite to 100 mL of blank urine, and then separating the spiked urine according to the described procedure. The collected fractions were evaporated to remove the solvents and most of the water at reduced pressure in a rotary evaporator (40 ^o^C), and then were lyophilized to remove traces of water and solvents with a freeze-dry system. The dried metabolites were then stored in a vacuum desiccator to further dry and prevent moisture absorption. After constant weights were reached, the purified metabolites were weighed using a Sartorius CP224S analytical balance (Sartorius AG, Goettingen, Germany).

### Purity analysis and structure elucidation

The purity of each peak fraction obtained by HSCCC was determined by NMR using internal standard method. The internal standard solution was prepared by dissolving a certain amount of hydroquinone with deuterated methanol (MeOD) giving a concentration of 0.02 mol/L. The fraction of about 1 mg was accurately weighed and dissolved in the hydroquinone internal standard solution. NMR spectra was recorded, the peak area ratio between the selected proton peak of the hydroquinone and that of the fraction was measured to calculate the purity of the fraction. Structural identification of each peak fraction was carried out by analyzing their MS and nuclear magnetic resonance (NMR) spectra. High resolution electrospray ionization MS (HR-ESI-MS) analyses were recorded with Agilent 6540 Q-TOF MS (Santa Clara, CA, USA). NMR spectra were recorded on Bruker Ascend 600 spectrometer equipped with cryogenic probe (Karlsruhe, Germany).

## Additional Information

**How to cite this article**: Yi, T. *et al*. HSCCC-based strategy for preparative separation of *in vivo* metabolites after administration of an herbal medicine: *Saussurea laniceps*, a case study. *Sci. Rep.* **6**, 33036; doi: 10.1038/srep33036 (2016).

## Supplementary Material

Supplementary Information

## Figures and Tables

**Table 1:** Partition coefficients (*K*) of the four metabolites in different two-phase solvent systems.

Solvent systems	Ratio of volume (v/v/v/v)	*K* values			
		M1	M2	M3	M4
MTBE–*n*-BuOH–ACN–H_2_O	2:2:1:5	0.05	0.06	0.13	0.15
	0:36:15:49	0.42	0.48	0.86	1.04
	10:30:11:49	0.65	0.80	1.20	1.46
	5:30:10:53	0.68	0.84	2.40	2.72
	3:32:10:55	1.24	1.36	2.60	2.80

**Table 2:** ^1^H and ^13^C NMR data for metabolites M1–M4 in MeOD (*δ* in ppm).

No.	M1		M2		M3		M4	
	*δ*_C_	*δ*_H_	*δ*_C_	*δ*_H_	*δ*_C_	*δ*_H_	*δ*_C_	*δ*_H_
2	163.2		163.5		162.9		163.3	
3	114.2	6.28 (1H, d, *J* = 9.5 Hz)	114.5	6.29 (1H, d, *J* = 9.5 Hz)	115.4	6.35 (1H, d, *J* = 9.5 Hz)	115.6	6.35 (1H, d, *J* = 9.5 Hz)
4	145.6	7.90 (1H, d, *J* = 9.5 Hz)	145.6	7.89 (1H, d, *J* = 9.5 Hz)	145.4	7.93 (1H, d, *J* = 9.5 Hz)	145.5	7.92 (1H, d, *J* = 9.5 Hz)
4a	115.3		114.6		116.9		116.6	
5	130.4	7.56 (1H, d, *J* = 8.6 Hz)	110.6	7.20 (1H, s)	130.0	7.60 (1H, d, *J* = 8.5 Hz)	111.1	7.23 (1H, s)
6	115.2	7.12 (1H, dd, *J* = 8.6, 2.3 Hz)	148.3		118.9	7.26 (1H, dd, *J* = 8.5, 2.2 Hz)	150.2	
7	162.3		151.8		157.3		146.8	
8	105.3	7.09 (1H, d, *J* = 2.3 Hz)	105.5	7.22 (1H, s)	109.6	7.33 (1H, d, *J* = 2.2 Hz)	110.8	7.55 (1H, s)
8a	156.7		150.7		156.1		149.7	
1′	101.9	5.06 (1H, d, *J* = 7.5 Hz)	101.9	5.10 (1H, d, *J* = 7.5 Hz)				
2′	74.6	3.54 (1H, m)	74.5	3.58 (1H, m)				
3′	73.5	3.54 (1H, m)	73.5	3.55 (1H, m)				
4′	77.6	3.54 (1H, m)	77.6	3.54 (1H, m)				
5′	76.5	3.83 (1H, m)	76.5	3.84 (1H, dd, *J* = 7.5, 2.1 Hz)				
6′	176.2		176.2					
OMe			57.0	3.91 (3H, s)			57.0	3.89 (3H, s)

NMR spectroscopy was performed on a Bruker Ascend 600 NMR spectrometer equipped with cryogenic probe (600 MHz for ^1^H NMR and 150 MHz for ^13^C NMR) using standard Bruker pulse programs. About 1 mg of each sample was dissolved in 0.5 ml MeOD with TMS as the internal standard (0.05% V/V), and the operation temperature was 298.0 K.
