# Supplementary material for: HSCCC-based strategy for preparative separation of in vivo metabolites after administration of an herbal medicine: Saussurea laniceps, a case study
Source: Sci Rep. 2016 Sep 13;6:33036. doi: 10.1038/srep33036 (PMC5020495; doi:10.1038/srep33036)
Supplement: Supplementary Information [file srep33036-s1.pdf]

HSCCC-based strategy for preparative separation of *in vivo* metabolites after administration of an herbal medicine: *Saussurea laniceps*, a case study

Tao Yi[1, 2], Lin Zhu[1], Guo-Yuan Zhu[3], Yi-Na Tang[1, 2], Jun Xu[1, 2], Jia-Yi Fan[1], Zhong-Zhen Zhao[1, 2] & Hu-Biao Chen[1, 2]

Affiliation

[1] School of Chinese Medicine, Hong Kong Baptist University, Kowloon Tong, Hong Kong Special Administrative Region, China;

[2] Institute of Research and Continuing Education (Shenzhen), Hong Kong Baptist University, Shenzhen, China;

[3] State Key Laboratory of Quality Research in Chinese Medicine, Macau University of Science and Technology, Taipa, Macau Special Administrative Region, China.

Corresponding

Correspondence and requests for materials should be addressed to T.Y. (yitao@hkbu.edu.hk) or H.B.C. (hbchen@hkbu.edu.hk)

Legends for Supplementary Information

Figure S1. ^{1}H-NMR spectrum of M1.

Figure S2. ^{13}C-NMR spectrum of M1.

Figure S3. ^{1}H-NMR spectrum of M2.

Figure S4. ^{13}C-NMR spectrum of M2.

Figure S5. ^{1}H-NMR spectrum of M3.

Figure S6. ^{13}C-NMR spectrum of M3.

Figure S7. ^{1}H-NMR spectrum of M4.

Figure S8. ^{13}C-NMR spectrum of M4.

Figure S9. +MS spectra (**A**) and +MS/MS spectra (**B**) of M1.

Figure S10. +MS spectra (**A**) and +MS/MS spectra (**B**) of M2.

Figure S11. +MS spectra (**A**) and +MS/MS spectra (**B**) of M3.

Figure S12. +MS spectra (**A**) and +MS/MS spectra (**B**) of M4.

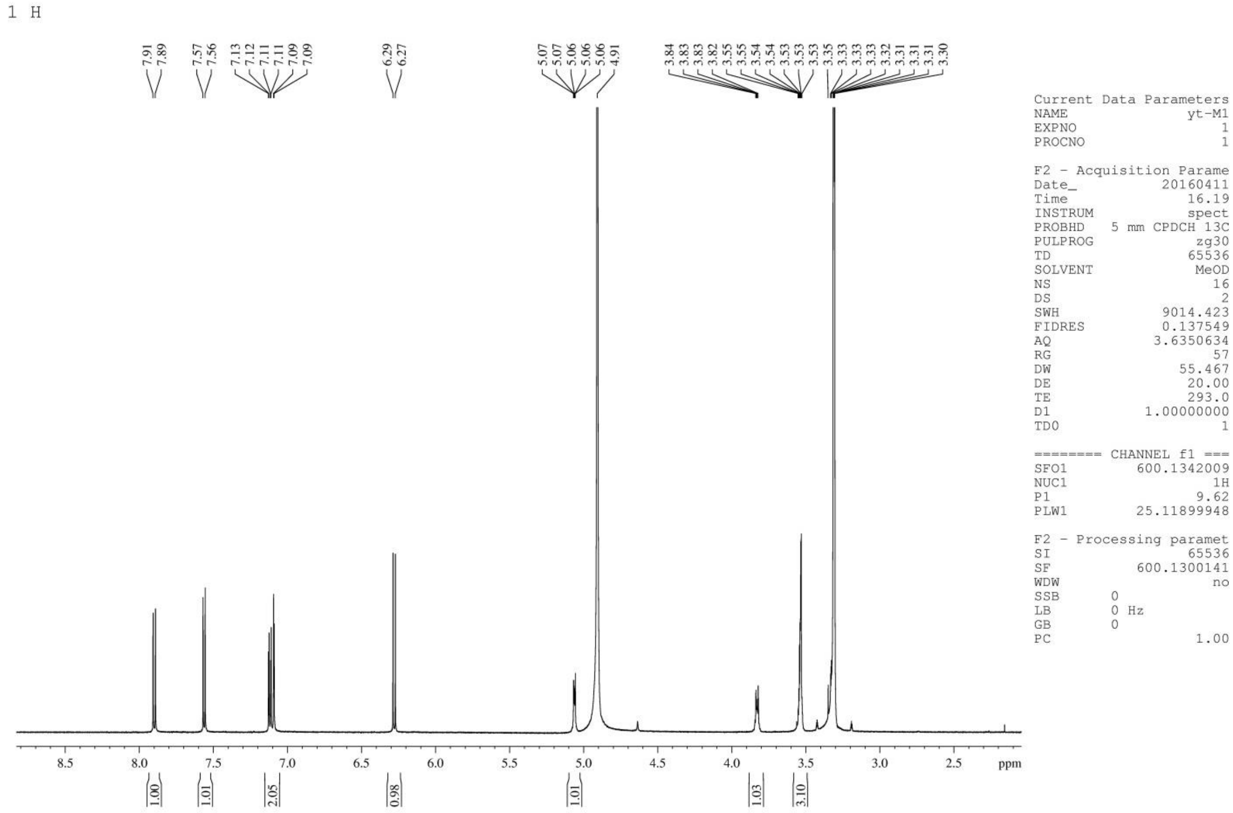

Figure S1. ^{1}H-NMR spectrum of M1.

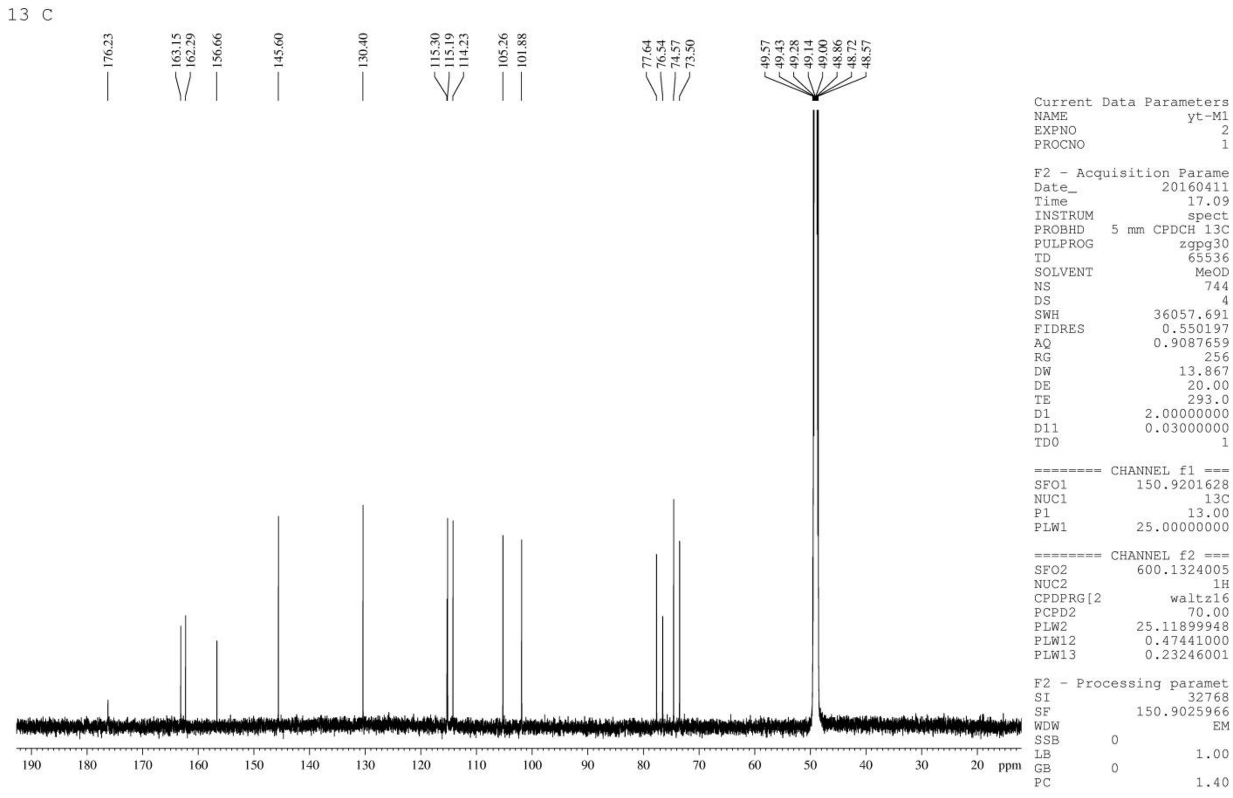

Figure S2. ^{13}C-NMR spectrum of M1.

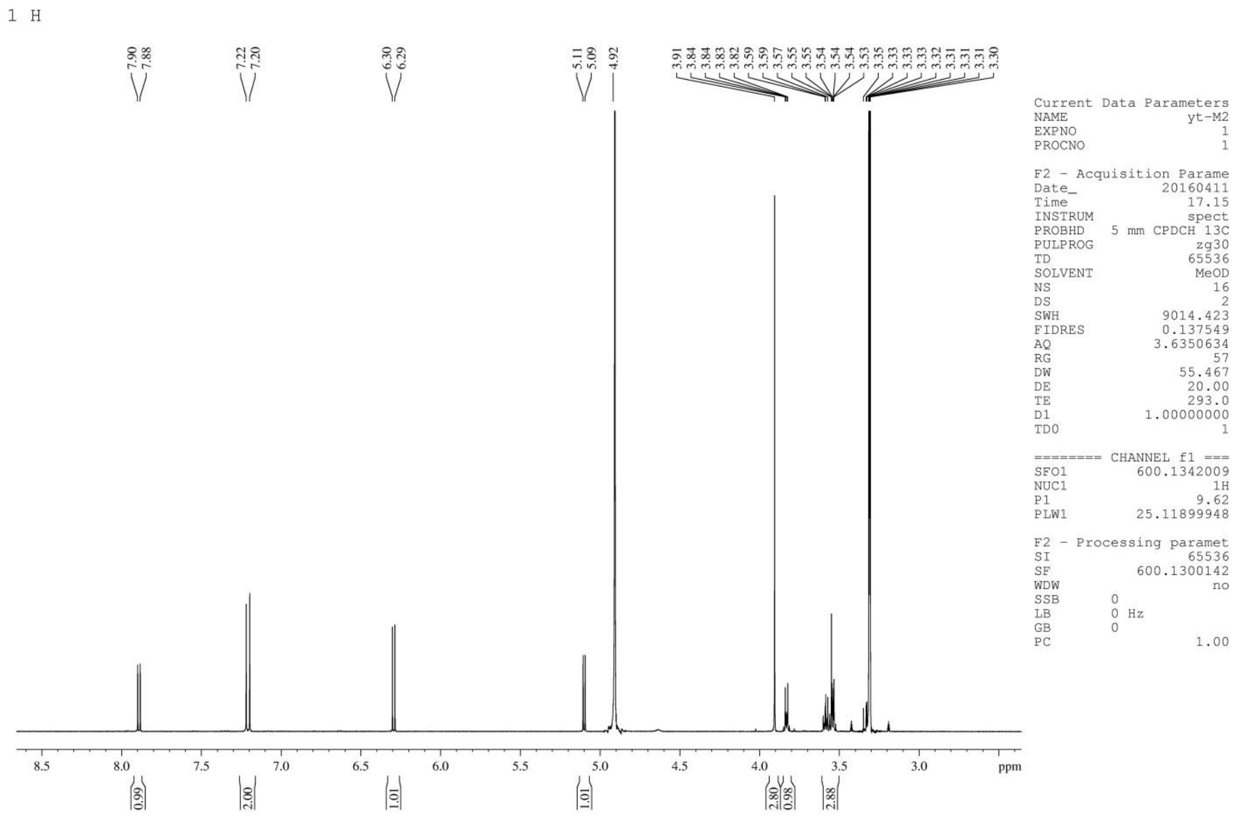

Figure S3. ^{1}H-NMR spectrum of M2.

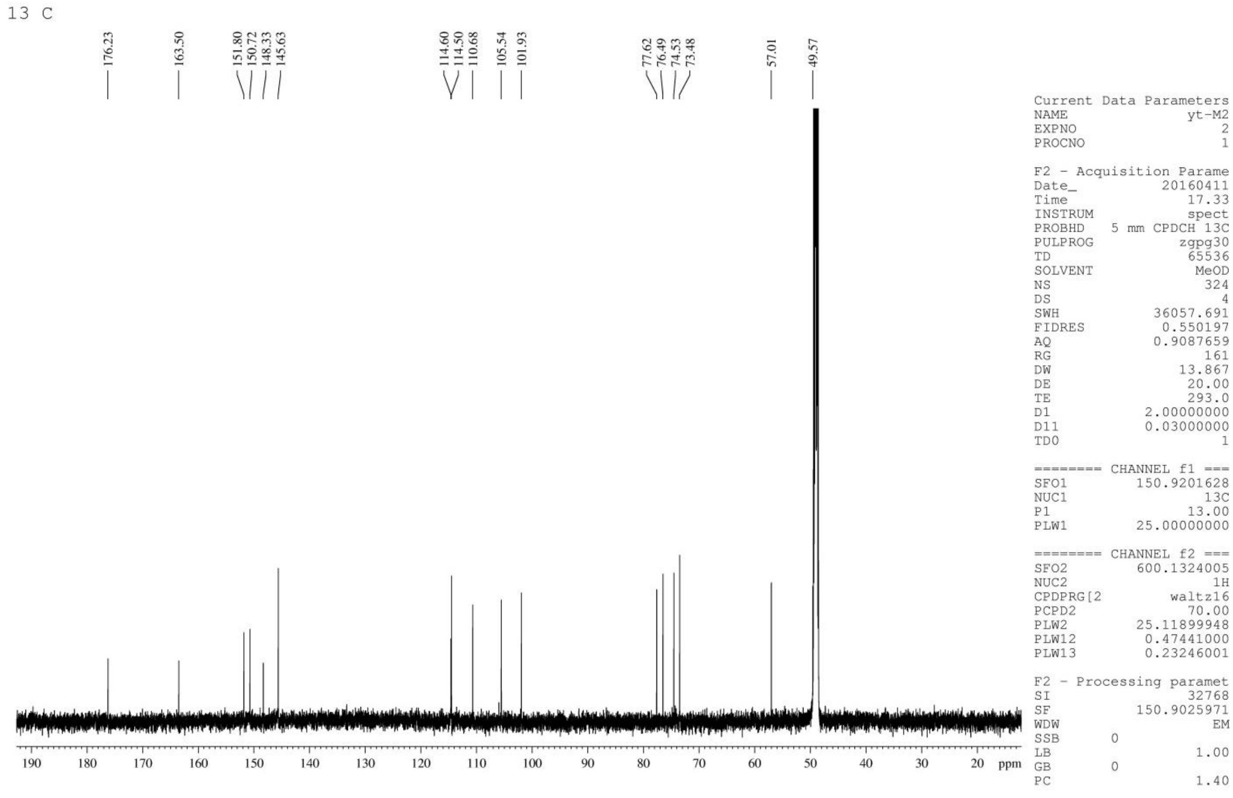

Figure S4. ^{13}C-NMR spectrum of M2.

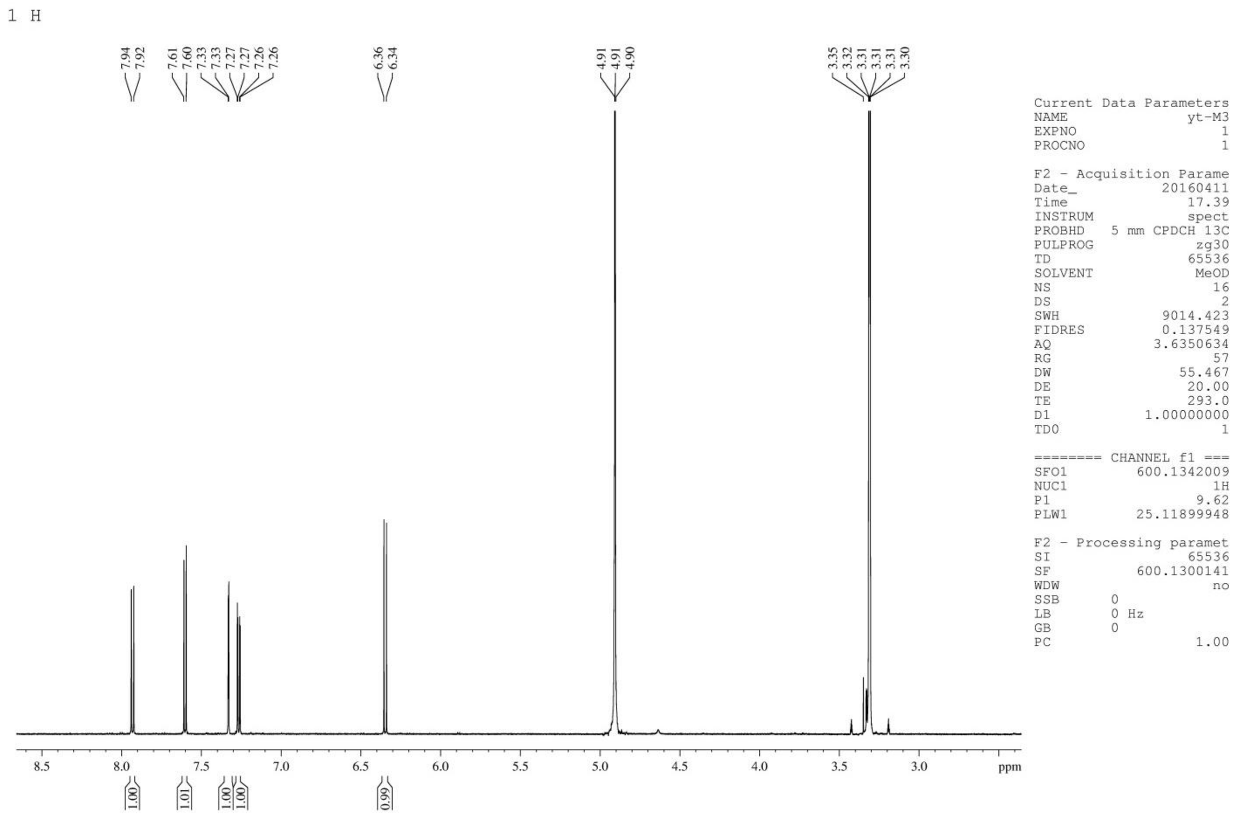

Figure S5. ^{1}H-NMR spectrum of M3.

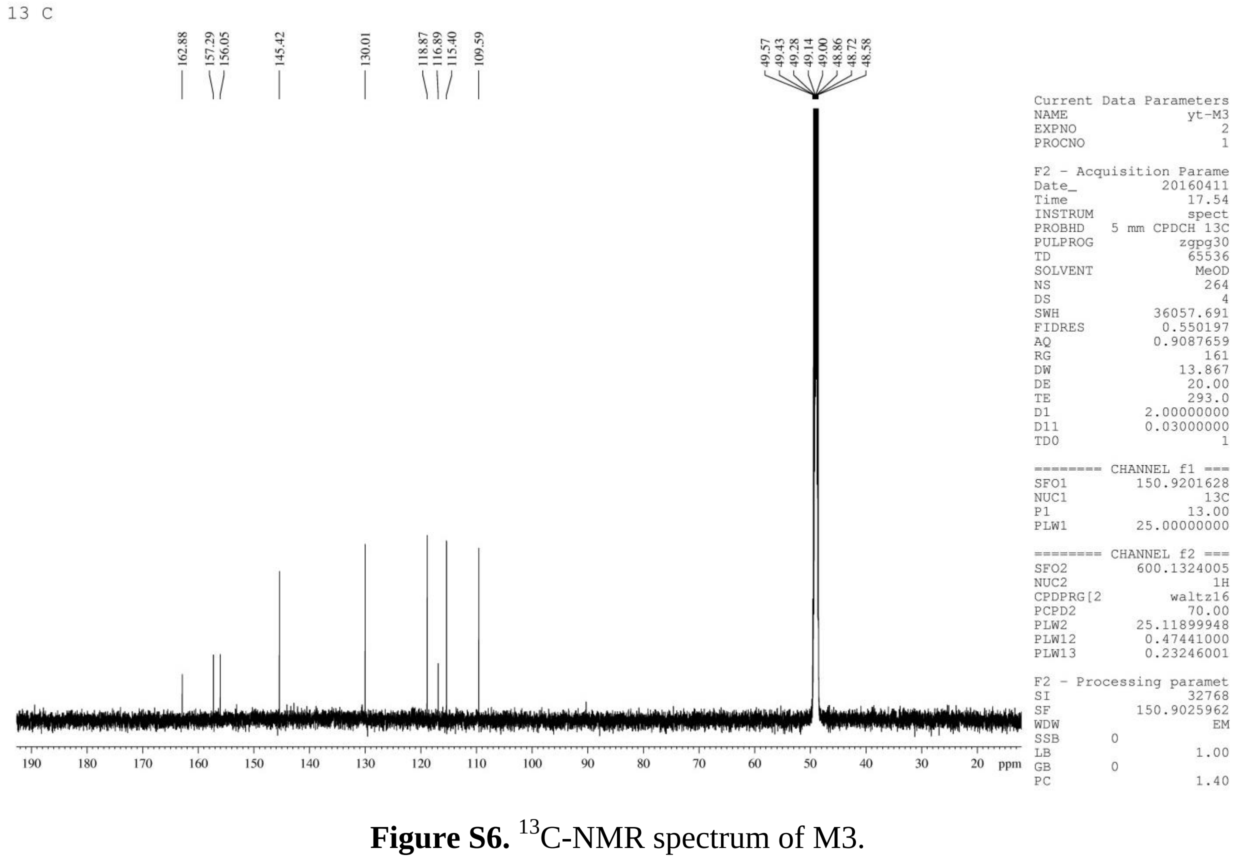

Figure S6. ^{13}C-NMR spectrum of M3.

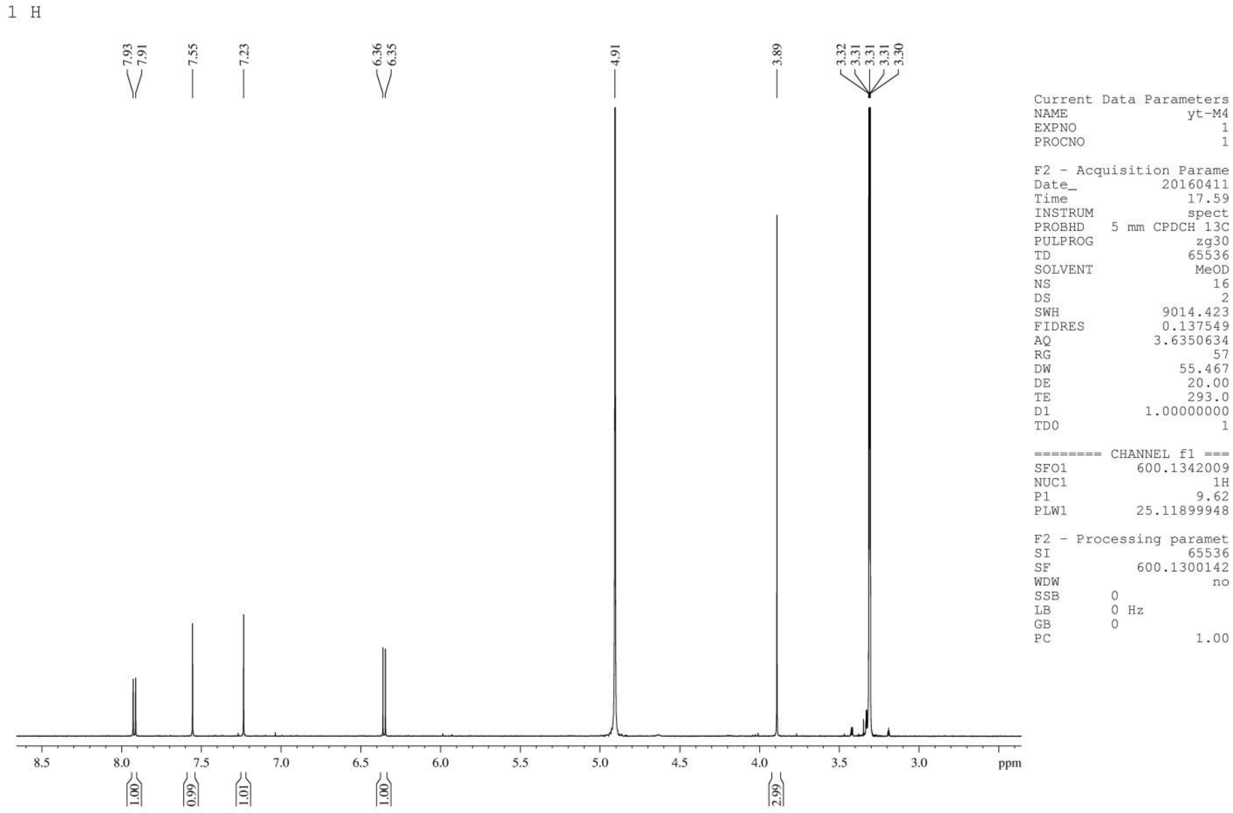

Figure S7. ^{1}H-NMR spectrum of M4.

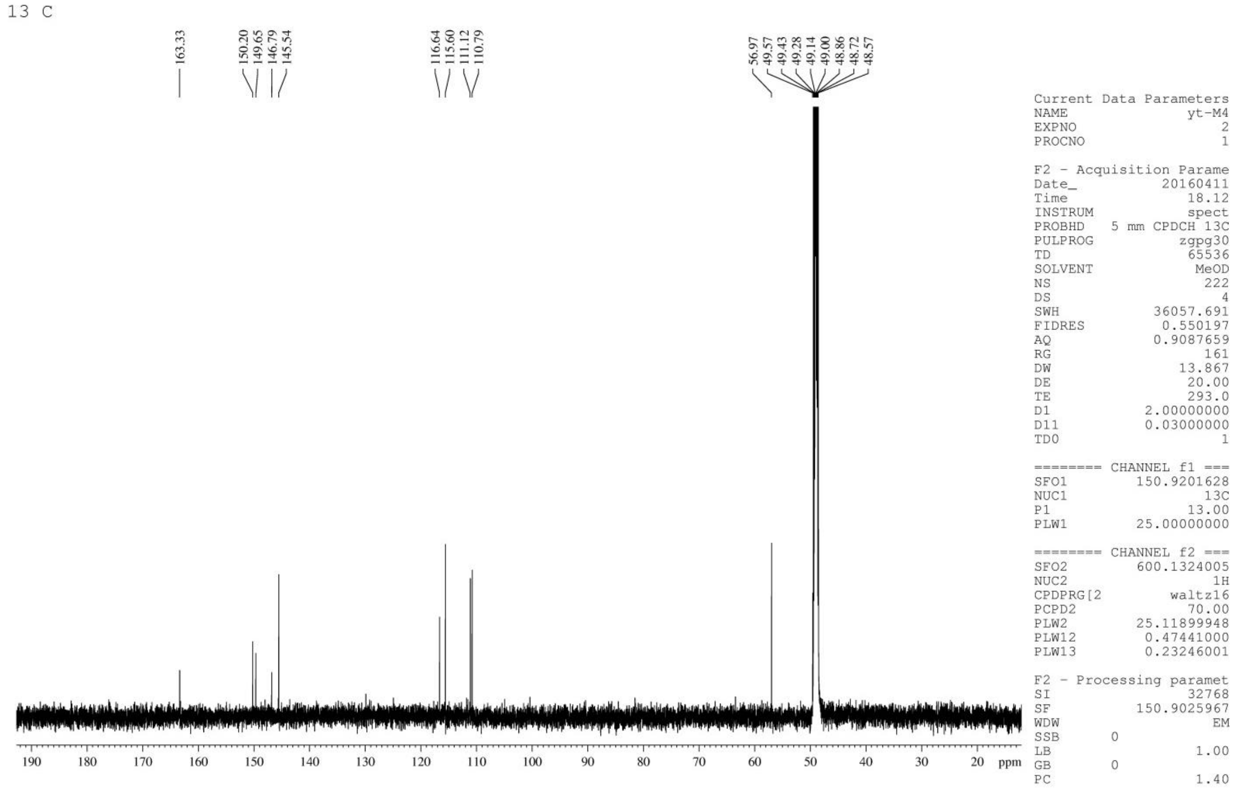

Figure S8. ^{13}C-NMR spectrum of M4.

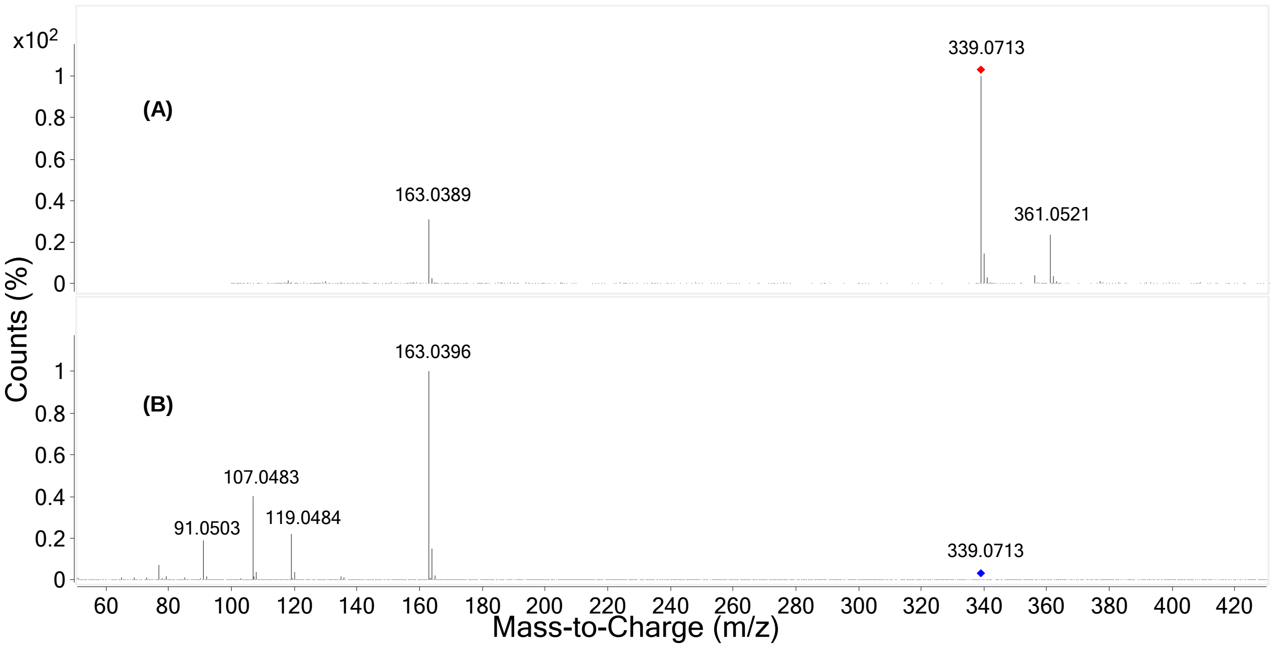

Figure S9. +MS spectra (**A**) and +MS/MS spectra (**B**) of M1.

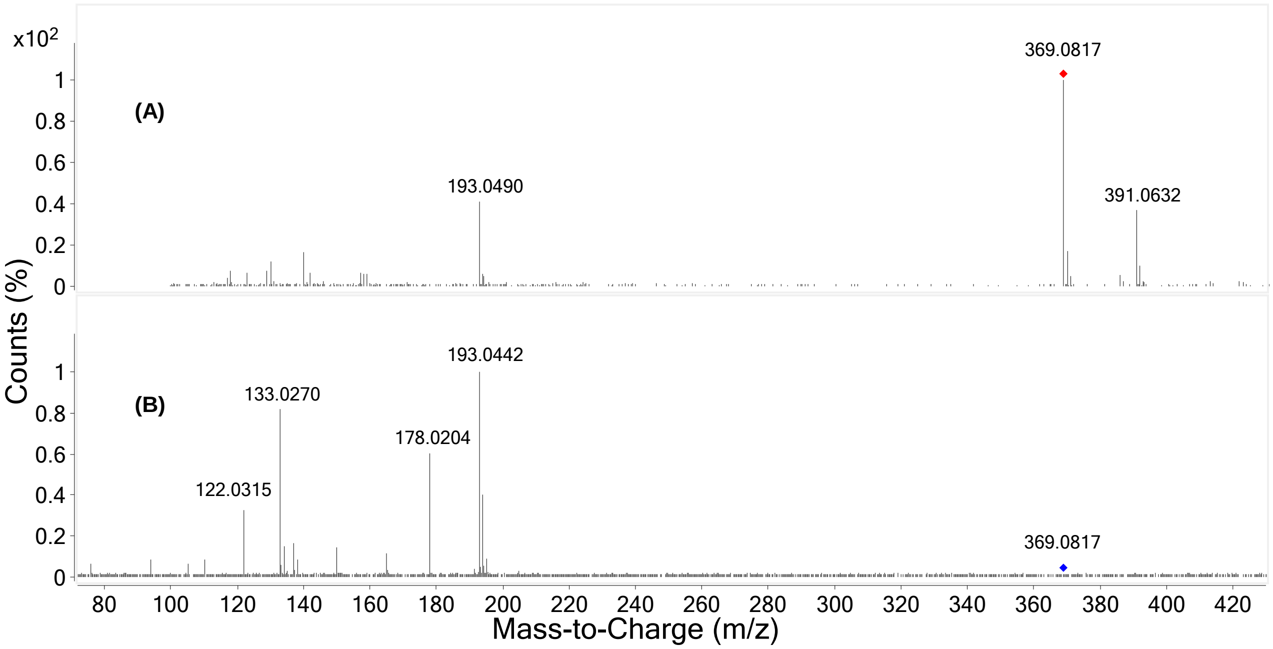

Figure S10. +MS spectra (**A**) and +MS/MS spectra (**B**) of M2.

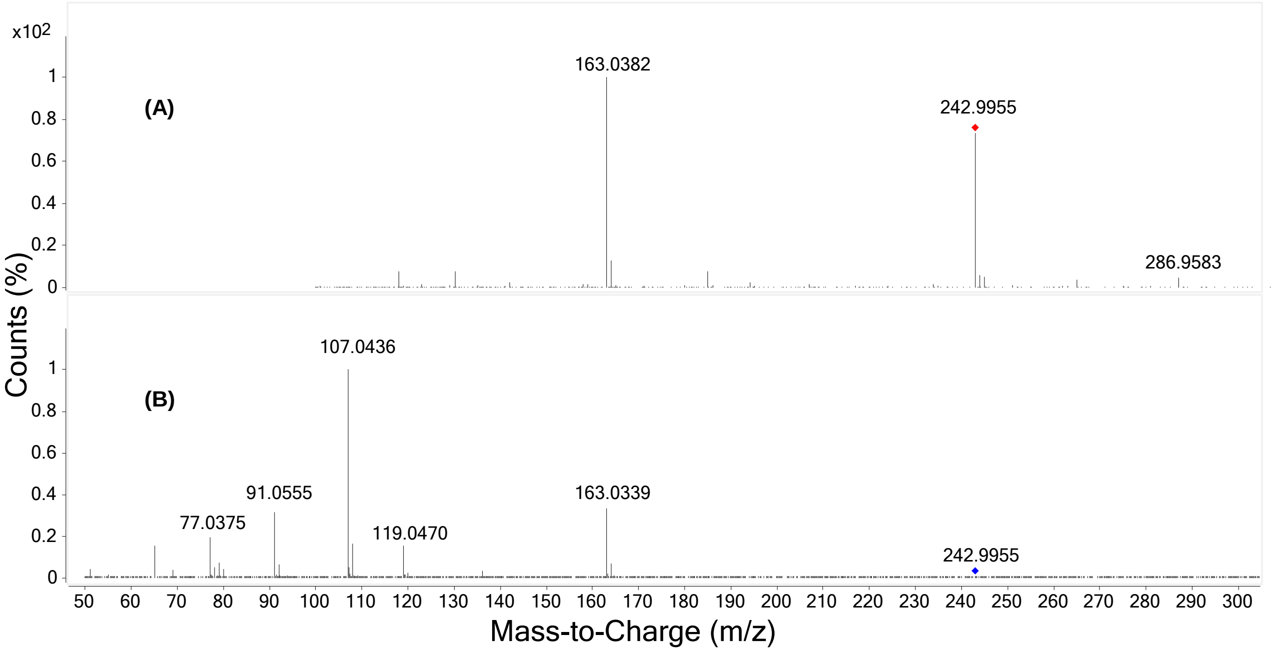

Figure S11. +MS spectra (**A**) and +MS/MS spectra (**B**) of M3.

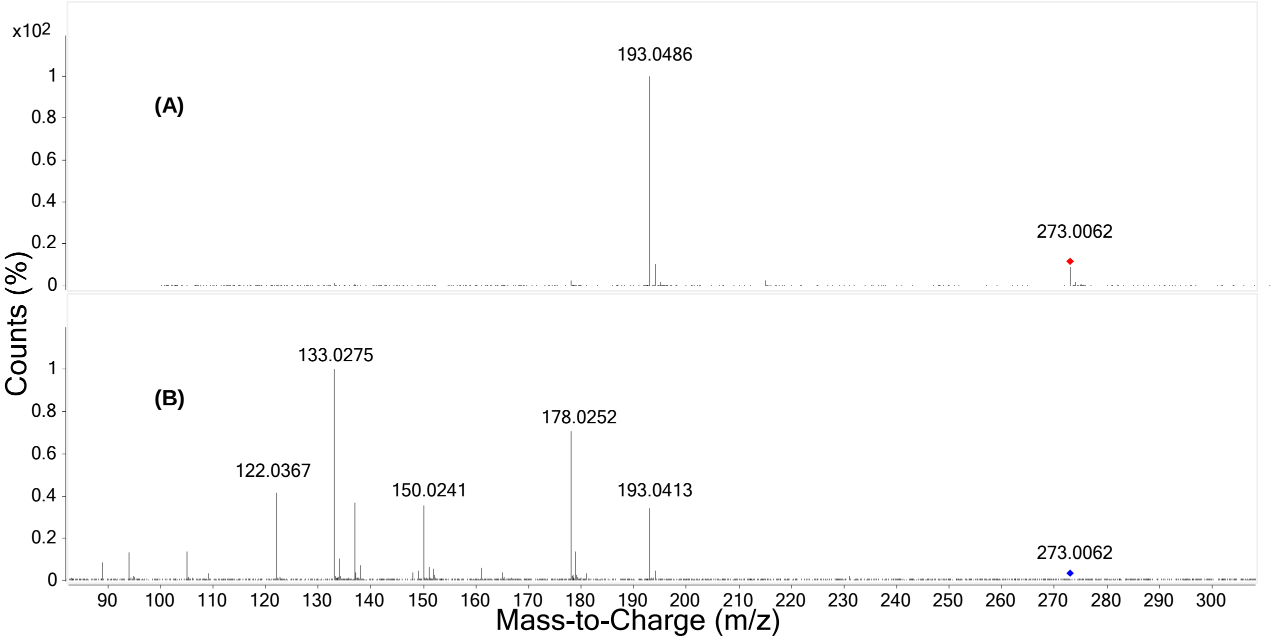

Figure S12. +MS spectra (**A**) and +MS/MS spectra (**B**) of M4.

(The end)
